# Supplementary material for: Variation in exposure in neighborhoods of Dhaka, Bangladesh across different environmental pathways: The influence of human behavior on fecal exposure in urban environments
Source: PLoS One. 2026 Jan 2;21(1):e0319883. doi: 10.1371/journal.pone.0319883 (PMC12758677; doi:10.1371/journal.pone.0319883)
Supplement: S3 Table — (DOCX) [file pone.0319883.s003.docx]

S3 Table: Frequency of reported child (age 5-12 years) behaviors (contact/ingestion) across nine environmental pathways based on household, community, and school surveys in Dhaka, Bangladesh (2017)

| Neighborhoods  and  Behavior frequency | Shared latrines  (Week)  n (%) | | Drain water (Month)  n (%) | Bathing water  (Week)  n (%) | Municipal drinking water^ǂ^ (Week)  n (%) | Non- municipal water^b^  (Week)  n (%) | | Surface water (Month)  n (%) | | | Produce  (Week)  n (%) | | Street food  (Week)  n (%) | | Flood water  (Month)  n (%) | |
| --- | --- | --- | --- | --- | --- | --- | --- | --- | --- | --- | --- | --- | --- | --- | --- | --- |
|  | **Community surveys, n=28** | | | | | | | | | | | | | | |  |
| **Floating communities**^#^ | | |  |  |  | |  | |  |  | |  | |  | | |
| > 10 times | 86 (81.1) | | 29 (64.4) | 74 (69.8) | 63 (69.2) | | 45 (42.1) | | 25 (53.2) | 59 (55.7) | | 65 (61.3) | | 24 (53.3) | | |
| 6-10 times | 6 (5.7) | | 5 (11.1) | 19 (17.9) | 1 (1.1) | | 4 (3.7) | | 1 (2.1) | 13 (12.3) | | 20 (18.9) | | 9 (20.0) | | |
| 1-5 times | 4 (3.8) | | 4 (8.9) | 4 (3.8) | 0 | | 1 (0.9) | | 3 (6.4) | 6 (5.7) | | 6 (5.7) | | 2 (4.4) | | |
| Never | 0 | | 4 (8.9) | 1 (0.9) | 23 (25.3) | | 53 (49.5) | | 11 (23.4) | 12 (11.3) | | 6 (5.7) | | 2 (4.4) | | |
| Not applicable^*^ | 10 (9.4) | | 3 (6.7) | 8 (7.5) | 4 (4.4) | | 4 (3.7) | | 7 (14.9) | 16 (15.1) | | 9 (8.5) | | 8 (17.8) | | |
| **Total**^§^ | **106 (100)** | | **45 (100)** | **106 (100)** | **91 (100)** | | **107 (100)** | | **47 (100)** | **106 (100)** | | **106 (100)** | | **45 (100)** | | |
| **Low-income** | | |  |  |  | |  | |  |  | |  | |  | | |
| > 10 times | | 127 (57.2) | 133 (59.9) | 143 (65.6) | 186 (84.2) | | 46 (20.8) | | 113 (52.8) | 116 (52.7) | | 137 (62.0) | | 157 (70.1) | | |
| 6-10 times | | 18 (8.1) | 38 (17.1) | 38 (17.4) | 13 (5.9) | | 18 (8.1) | | 21 (9.8) | 36 (16.4) | | 43 (19.5) | | 12 (5.4) | | |
| 1-5 times | | 36 (16.2) | 10 (4.5) | 14 (6.4) | 4 (1.8) | | 11 (5.0) | | 12 (5.6) | 28 (12.7) | | 22 (10.0) | | 19 (8.5) | | |
| Never | | 30 (13.5) | 24 (10.8) | 7 (3.2) | 13 (5.9) | | 125 (56.6) | | 34 (15.9) | 30 (13.6) | | 16 (7.2) | | 24 (10.7) | | |
| Not applicable^*^ | | 11 (5.0) | 17 (7.7) | 16 (7.3) | 5 (2.3) | | 21 (9.5) | | 34 (15.9) | 10 (4.5) | | 3 (1.4) | | 12 (5.4) | | |
| **Total**^§^ | | **222 (100)** | **222 (100)** | **218 (100)** | **221 (100)** | | **221 (100)** | | **214 (100)** | **220 (100)** | | **221 (100)** | | **224 (100)** | | |
| **High-income** | |  | **Data not collected** | | | | | | | | | | | | | |
|  | | **School surveys, n=28** | | | | | | | | | | | | | | |
| **Floating communities**^#^ | | |  |  |  | |  | |  |  | |  | |  | | |
| > 10 times | | 39 (32.8) | 49 (41.9) | 66 (55.5) | 92 (77.3) | | 51 (42.9) | | 30 (25.2) | 41 (34.5) | | 63 (52.9) | | 46 (38.7) | | |
| 6-10 times | | 23 (19.3) | 10 (8.5) | 46 (38.7) | 9 (7.6) | | 8 (6.7) | | 26 (21.8) | 33 (27.7) | | 23 (19.3) | | 23 (19.3) | | |
| 1-5 times | | 6 (5.0) | 22 (18.8) | 5 (4.2) | 8 (6.7) | | 12 (10.1) | | 22 (18.5) | 18 (15.1) | | 15 (12.6) | | 27 (22.7) | | |
| Never | | 41 (34.5) | 25 (21.4) | 0 | 8 (6.7) | | 44 (37.0) | | 32 (26.9) | 20 (16.8) | | 14 (11.8) | | 18 (15.1) | | |
| Not applicable^*^ | | 10 (8.4) | 11 (9.4) | 2 (1.7) | 2 (1.7) | | 4 (3.4) | | 9 (7.6) | 7 (5.9) | | 4 (3.4) | | 5 (4.2) | | |
| **Total**^§^ | | **119 (100)** | **117 (100)** | **119 (100)** | **119 (100)** | | **119 (100)** | | **119 (100)** | **119 (100)** | | **119 (100)** | | **119 (100)** | | |
| **Low-income** | |  |  |  |  | |  | |  |  | |  | |  | | |
| > 10 times | | 106 (39.1) | 121 (44.8) | 97 (35.9) | 187 (69.5) | | 71 (26.2) | | 72 (27.0) | 75 (27.7) | | 110 (40.6) | | 101 (37.3) | | |
| 6-10 times | | 50 (18.5) | 45 (16.7) | 106 (39.3) | 30 (11.2) | | 32 (11.8) | | 42 (15.7) | 71 (26.2) | | 59 (21.8) | | 57 (21.0) | | |
| 1-5 times | | 49 (18.1) | 53 (19.6) | 54 (20.0) | 16 (5.9) | | 50 (18.5) | | 54 (20.2) | 74 (27.3) | | 54 (19.9) | | 69 (25.5) | | |
| Never | | 56 (20.7) | 45 (16.7) | 1 (0.4) | 26 (9.7) | | 98 (36.2) | | 94 (35.2) | 38 (14.0) | | 34 (12.5) | | 32 (11.8) | | |
| Not applicable^*^ | | 10 (3.7) | 6 (2.2) | 12 (4.4) | 10 (3.7) | | 20 (7.4) | | 5 (1.9) | 13 (4.8) | | 14 (5.2) | | 12 (4.4) | | |
| **Total**^§^ | | **271 (100)** | **270 (100)** | **270 (100)** | **269 (100)** | | **271 (100)** | | **267 (100)** | **271 (100)** | | **271 (100)** | | **271 (100)** | | |
| **High-income** | |  |  |  |  | |  | |  |  | |  | |  | | |
| > 10 times | | 44 (21.3) | 77 (37.2) | 44 (21.3) | 158 (76.3) | | 54 (26.1) | | 31 (15.0) | 52 (25.1) | | 75 (36.2) | | 49 (23.7) | | |
| 6-10 times | | 28 (13.5) | 35 (16.9) | 127 (61.4) | 15 (7.2) | | 24 (11.6) | | 27 (13.0) | 36 (17.4) | | 18 (8.7) | | 40 (19.3) | | |
| 1-5 times | | 15 (7.2) | 25 (12.1) | 23 (11.1) | 8 (3.9) | | 32 (15.5) | | 32 (15.5) | 66 (31.9) | | 61 (29.5) | | 72 (34.8) | | |
| Never | | 113 (54.6) | 65 (31.4) | 3 (1.4) | 23 (11.1) | | 83 (40.1) | | 108 (52.2) | 44 (21.3) | | 41 (19.8) | | 39 (18.8) | | |
| Not applicable^*^ | | 7 (3.4) | 5 (2.4) | 10 (4.8) | 3 (1.4) | | 14 (6.8) | | 9 (4.3) | 9 (4.3) | | 12 (5.8) | | 7 (3.4) | | |
| **Total**^§^ | | **207 (100)** | **207 (100)** | **207 (100)** | **207 (100)** | | **207 (100)** | | **207 (100)** | **207 (100)** | | **207 (100)** | | **207 (100)** | | |

^*^Not applicable=Unable to collect during surveys

^ǂ^For community and school surveys, the total number of responses was sometimes smaller than the total number of participants as these surveys were conducted in a group setting and participants may have left the survey early or left the survey temporarily

^#^For community and school surveys, the total number of responses was sometimes smaller than the total number of participants as these surveys were conducted in a group setting and participants may have left the survey early or left the survey temporarily

^§^Certain pathways were defined as “not applicable” for one of the floating communities and therefore those questions were skipped by enumerators while administering the survey.
