# Supplementary material for: Variation in exposure in neighborhoods of Dhaka, Bangladesh across different environmental pathways: The influence of human behavior on fecal exposure in urban environments
Source: PLoS One. 2026 Jan 2;21(1):e0319883. doi: 10.1371/journal.pone.0319883 (PMC12758677; doi:10.1371/journal.pone.0319883)
Supplement: S4 Table — (DOCX) [file pone.0319883.s004.docx]

S4 Table: Frequency of reported adult behaviors (contact/ingestion) across nine environmental pathways based on household, community and school surveys in Dhaka, Bangladesh (2017)

| Neighborhoods  and  Behavior frequency | Shared latrines  (Week)  n (%) | Drain water (Month)  n (%) | Bathing water  (Week)  n (%) | Municipal drinking water (Week)  n (%) | Non- municipal water  (Week)  n (%) | Surface water (Month)  n (%) | Produce  (Week)  n (%) | Street food  (Week)  n (%) | Flood water  (Month)  n (%) |
| --- | --- | --- | --- | --- | --- | --- | --- | --- | --- |
|  |  |  | **Household surveys, n=823)** | | | | |  |  |
| **Floating communities (n=200)**^†^ | | | |  |  |  |  |  |  |
| > 10 times | 132 (66.0) | 95 (47.5) | 63 (31.5) | 86 (43.0) | 108 (54.0) | 63 (31.5) | 43 (21.5) | 19 (9.5) | 124 (62.0) |
| 6-10 times | 60 (30.0) | 29 (14.5) | 134 (67.0) | 0 | 10 (5.0) | 15 (7.5) | 56 (28.0) | 41 (20.5) | 18 (9.0) |
| 1-5 times | 4 (2.0) | 12 (6.0) | 3 (1.5) | 0 | 10 (5.0) | 10 (5.0) | 84 (42.0) | 93 (46.5) | 16 (8.0) |
| Never | 3 (1.5) | 36 (18.0) | 0 | 109 (54.5) | 49 (24.5) | 88 (44.0) | 15 (7.5) | 44 (22.0) | 18 (9.0) |
| I don’t know | 0 | 0 | 0 | 0 | 0 | 0 | 1 (0.5) | 0 | 0 |
| Not applicable^*^ | 1 (0.5) | 28 (14.0) | 0 | 5 (2.5) | 23 (11.5) | 24 (12.0) | 1 (0.5) | 3 (1.5) | 24 (12.0) |
| **Low-income (n=401)** |  |  |  |  |  |  |  |  |  |
| > 10 times | 240 (59.9) | 230 (57.4) | 113 (28.2) | 262 (65.3) | 62 (15.5) | 102 (25.4) | 54 (13.5) | 34 (8.5) | 206 (51.4) |
| 6-10 times | 99 (24.7) | 56 (14.0) | 263 (65.6) | 8 (2.0) | 18 (4.5) | 31 (7.7) | 94 (23.4) | 55 (13.7) | 56 (14.0) |
| 1-5 times | 21 (5.2) | 55 (13.7) | 25 (6.2) | 1 (0.2) | 53 (13.2) | 52 (13.0) | 218 (54.4) | 193 (48.1) | 116 (28.9) |
| Never | 41 (10.2) | 58 (14.5) | 0 | 130 (32.4) | 261 (65.1) | 209 (52.1) | 32 (8.0) | 117 (29.2) | 15 (3.7) |
| I don’t know | 0 | 0 | 0 | 0 | 0 | 0 | 3 (0.7) | 1 (0.2) | 1 (0.2) |
| Not applicable | 0 | 2 (0.5) | 0 | 0 | 7 (1.7) | 7 (1.7) | 0 | 1 (0.2) | 7 (1.7) |
| **High-income (n=222)** |  |  |  |  |  |  |  |  |  |
| > 10 times | 2 (0.9) | 12 (5.4) | 10 (4.5) | 143 (64.4) | 9 (4.1) | 4 (1.8) | 9 (4.1) | 7 (3.2) | 6 (2.7) |
| 6-10 times | 2 (0.9) | 3 (1.4) | 153 (68.9) | 12 (5.4) | 6 (2.7) | 4 (1.8) | 59 (26.6) | 5 (2.3) | 9 (4.1) |
| 1-5 times | 8 (3.6) | 9 (4.1) | 22 (9.9) | 8 (3.6) | 21 (9.5) | 17 (7.7) | 105 (47.3) | 92 (41.4) | 56 (25.2) |
| Never | 141 (63.5) | 154 (69.4) | 3 (1.4) | 10 (4.5) | 129 (58.1) | 142 (64.0) | 7 (3.2) | 75 (33.8) | 107 (48.2) |
| I don’t know | 28 (12.6) | 13 (5.9) | 4 (1.8) | 15 (6.8) | 19 (8.6) | 23 (10.4) | 8 (3.6) | 5 (2.3) | 9 (4.1) |
| Not applicable | 41 (18.5) | 31 (14.0) | 30 (13.5) | 34 (15.3) | 38 (17.1) | 32 (14.4) | 34 (15.3) | 38 (17.1) | 35 (15.8) |
| **Community surveys, n=823)** | | | | | | | | | |
| **Floating communities**^†^ | |  |  |  |  |  |  |  |  |
| > 10 times | 123 (72.8) | 39 (46.4) | 55 (32.4) | 93 (61.2) | 82 (48.5) | 36 (42.9) | 78 (45.9) | 87 (51.2) | 56 (66.7) |
| 6-10 times | 9 (5.3) | 1 (1.2) | 59 (34.7) | 4 (2.6) | 2 (1.2) | 6 (7.1) | 16 (9.4) | 24 (14.1) | 4 (4.8) |
| 1-5 times | 11 (6.5) | 7 (8.3) | 27 (15.9) | 1 (0.7) | 2 (1.2) | 7 (8.3) | 17 (10.0) | 31 (18.2) | 4 (4.8) |
| Never | 4 (2.4) | 20 (23.8) | 1 (0.6) | 33 (21.7) | 71 (42.0) | 26 (31.0) | 32 (18.8) | 8 (4.7) | 4 (4.8) |
| Not applicable^*^ | 22 (13.0) | 17 (20.2) | 28 (16.5) | 21 (13.8) | 12 (7.1) | 9 (10.7) | 27 (15.9) | 20 (11.8) | 16 (19.0) |
| **Total** ^††^ | **169 (100)** | **84 (100)** | **170 (100)** | **152 (100)** | **169 (100)** | **84 (100)** | **170 (100)** | **170 (100)** | **84 (100)** |
| **Low-income (n=)** |  |  |  |  |  |  |  |  |  |
| > 10 times | 205 (67.7) | 192 (59.4) | 99 (32.0) | 250 (79.4) | 70 (22.5) | 106 (34.4) | 183 (60.0) | 111 (36.2) | 222 (69.4) |
| 6-10 times | 32 (10.6) | 26 (8.0) | 142 (46.0) | 11 (3.5) | 39 (12.5) | 22 (7.1) | 38 (12.5) | 42 (13.7) | 23 (7.2) |
| 1-5 times | 19 (6.3) | 32 (9.9) | 25 (8.1) | 4 (1.3) | 12 (3.9) | 40 (13.0) | 32 (10.5) | 67 (21.8) | 25 (7.8) |
| Never | 40 (13.2) | 45 (13.9) | 9 (2.9) | 31 (9.8) | 177 (56.9) | 107 (34.7) | 31 (10.2) | 64 (20.8) | 20 (6.2) |
| Not applicable^*^ | 7 (2.3) | 28 (8.7) | 34 (11.0) | 19 (6.0) | 13 (4.2) | 33 (10.7) | 21 (6.9) | 23 (7.5) | 30 (9.4) |
| **Total**^††^ | **303 (100)** | **323 (100)** | **309 (100)** | **315 (100)** | **311 (100)** | **308 (100)** | **305 (100)** | **307 (100)** | **320 (100)** |
|  |  |  | **School surveys, N=35 (597)** | | | | |  |  |
| **Floating communities**^†^ | |  |  |  |  |  |  |  |  |
| > 10 times | 37 (31.1) | 44 (37.0) | 58 (48.7) | 89 (74.8) | 30 (25.2) | 23 (19.3) | 43 (36.1) | 32 (26.9) | 45 (37.8) |
| 6-10 times | 15 (12.6) | 16 (13.4) | 54 (45.4) | 11 (9.2) | 22 (18.5) | 18 (15.1) | 28 (23.5) | 22 (18.5) | 25 (21.0) |
| 1-5 times | 15 (12.6) | 15 (12.6) | 2 (1.7) | 5 (4.2) | 11 (9.2) | 23 (19.3) | 15 (12.6) | 21 (17.6) | 20 (16.8) |
| Never | 38 (31.9) | 26 (21.8) | 0 | 7 (5.9) | 37 (31.1) | 43 (36.1) | 15 (12.6) | 28 (23.5) | 11 (9.2) |
| Not applicable^*^ | 14 (11.8) | 18 (15.1) | 5 (4.2) | 7 (5.9) | 19 (16.0) | 12 (10.1) | 18 (15.1) | 16 (13.4) | 18 (15.1) |
| **Total**^††^ | **119 (100)** | **119 (100)** | **119 (100)** | **119 (100)** | **119 (100)** | **119 (100)** | **119 (100)** | **119 (100)** | **119 (100)** |
| **Low-income (n=)** |  |  |  |  |  |  |  |  |  |
| > 10 times | 90 (33.2) | 106 (39.4) | 88 (32.5) | 185 (68.8) | 45 (16.6) | 53 (19.6) | 70 (25.8) | 49 (18.1) | 99 (37.8) |
| 6-10 times | 63 (23.2) | 42 (15.6) | 123 (45.4) | 32 (11.9) | 38 (14.0) | 47 (17.3) | 54 (19.9) | 42 (15.5) | 53 (20.2) |
| 1-5 times | 36 (13.3) | 48 (17.8) | 39 (14.4) | 17 (6.3) | 50 (18.5) | 67 (24.7) | 79 (29.2) | 58 (21.4) | 50 (19.1) |
| Never | 53 (19.6) | 52 (19.3) | 0 | 25 (9.3) | 94 (34.7) | 89 (32.8) | 36 (13.3) | 77 (28.4) | 26 (9.9) |
| Not applicable^*^ | 29 (10.7) | 21 (7.8) | 21 (7.7) | 10 (3.7) | 44 (16.2) | 15 (5.5) | 32 (11.8) | 45 (16.6) | 34 (13.0) |
| **Total**^††^ | **271 (100)** | **269 (100)** | **271 (100)** | **269 (100)** | **271 (100)** | **271 (100)** | **271(100)** | **271 (100)** | **262 (100)** |
| **High-income** |  |  |  |  |  |  |  |  |  |
| > 10 times | 30 (14.5) | 67 (32.4) | 73 (35.4) | 153 (73.9) | 49 (23.7) | 37 (17.9) | 47 (22.7) | 29 (14.1) | 50 (24.2) |
| 6-10 times | 16 (7.7) | 29 (14.0) | 104 (50.5) | 16 (7.7) | 24 (11.6) | 27 (13.0) | 40 (19.3) | 33 (16.0) | 44 (21.3) |
| 1-5 times | 13 (6.3) | 34 (16.4) | 21 (10.2) | 8 (3.9) | 31 (15.0) | 46 (22.2) | 50 (24.2) | 40 (19.4) | 54 (26.1) |
| Never | 125 (60.4) | 61 (29.5) | 0 | 25 (12.1) | 78 (37.7) | 83 (40.1) | 36 (17.4) | 77 (37.4) | 34 (16.4) |
| Not applicable^*^ | 23 (11.1) | 16 (7.7) | 8 (3.9) | 5 (2.4) | 25 (12.1) | 14 (6.8) | 34 (16.4) | 27 (13.1) | 25 (12.1) |

^*^Not applicable=Unable to collect during surveys

^†^For community and school surveys, the total number of responses was sometimes smaller than the total number of participants as these surveys were conducted in a group setting and participants may have left the survey early or left the survey temporarily

^††^For community and school surveys, the total number of responses was sometimes smaller than the total number of participants as these surveys were conducted in a group setting and participants may have left the survey early or left the survey temporarily

^§^Certain pathways were defined as “not applicable” for one of the floating communities and therefore those questions were skipped by enumerators while administering the survey.
