# Supplementary material for: Variation in exposure in neighborhoods of Dhaka, Bangladesh across different environmental pathways: The influence of human behavior on fecal exposure in urban environments
Source: PLoS One. 2026 Jan 2;21(1):e0319883. doi: 10.1371/journal.pone.0319883 (PMC12758677; doi:10.1371/journal.pone.0319883)
Supplement: S5 Table — (DOCX) [file pone.0319883.s005.docx]

S5 Table: Summary of estimated monthly *E. coli* doses (log10 MPN) for adults by exposure pathway and neighborhood: Dose-Adults

| **Neighborhoods** | **Shared latrines** | **Drain water** | **Bathing water** | **Municipal drinking water^§^** | **Non- municipal water^§^** | **Surface water** | **Produce** | **Street food** | **Flood water** |
| --- | --- | --- | --- | --- | --- | --- | --- | --- | --- |
| Badda | 1.9900 | 6.75 | 1.89 | 4.76 | 5.09 | 6.96 | 6.91 | 5.76 | 4.24 |
| Dhanmondi | 0.1290 | 7.07 | 4.00 | 5.50 | 3.32 | 3.91 | 6.90 | 7.31 | 5.57 |
| Gabtoli | 1.5200 | 6.48 | 1.36 | 3.36 | 3.25 | 8.16 | 7.16 | 7.38 | 6.96 |
| Gulshan | -0.00969 | 4.41 | 1.84 | 3.23 | 1.46 | 5.13 | 6.48 | 4.35 | 3.12 |
| Hazaribagh | 3.2300 | 6.15 | 3.60 | 8.52 | 6.32 | 6.27 | 4.80 | 4.91 | 5.70 |
| Kalshi | 1.4100 | 6.17 | 3.04 | 3.85 | 5.56 | 5.90 | 6.40 | 6.17 | 4.37 |
| Kamalapur | 3.5300 | 6.89 | 3.80 | 8.23 | 4.24 | 7.26 | 7.44 | 5.73 | 5.30 |
| Motijhil | 1.8300 | 6.25 | 2.72 | 6.68 | 3.65 | 2.76 | 5.72 | 7.17 | 4.53 |
| Shampur | 0.8050 | 6.46 | 5.38 | 8.57 | 2.62 | 6.06 | 7.50 | 3.86 | 5.65 |
| Uttarkhan | 2.5000 | 5.78 | 3.98 | 7.40 | 2.61 | 5.58 | 6.01 | 7.21 | 3.62 |

^*^Municipal drinking water' refers to the water supplied by the city government

^§^Non-municipal water' includes water from private sources such as wells or boreholes
