# Supplementary material for: Variation in exposure in neighborhoods of Dhaka, Bangladesh across different environmental pathways: The influence of human behavior on fecal exposure in urban environments
Source: PLoS One. 2026 Jan 2;21(1):e0319883. doi: 10.1371/journal.pone.0319883 (PMC12758677; doi:10.1371/journal.pone.0319883)
Supplement: S6 Table — (DOCX) [file pone.0319883.s006.docx]

S6 Table: Summary of estimated monthly *E. coli* doses (log10 MPN) for children by exposure pathway and neighborhood: Dose- Children

| **Neighborhoods** | **Shared latrines** | **Drain water** | **Bathing water** | **Municipal drinking water**^*^ | **Non- municipal water^§^** | **Surface water** | **Produce** | **Street food** | **Flood water** |
| --- | --- | --- | --- | --- | --- | --- | --- | --- | --- |
| Badda | 1.850 | 8.28 | 2.15 | 4.31 | 4.28 | 8.35 | 6.43 | 5.81 | 5.40 |
| Dhanmondi | 0.289 | 9.44 | 3.80 | 5.10 | 2.91 | 4.77 | 7.50 | 7.21 | 6.09 |
| Gabtoli | 1.210 | 8.23 | 1.75 | 2.76 | 2.83 | 9.11 | 6.49 | 6.91 | 9.06 |
| Gulshan | 0.360 | 5.89 | 1.54 | 2.88 | 1.50 | 5.25 | 7.21 | 4.16 | 4.28 |
| Hazaribagh | 2.610 | 7.33 | 4.04 | 8.20 | 4.54 | 7.06 | 4.22 | 4.55 | 7.00 |
| Kalshi | 1.490 | 6.89 | 3.62 | 3.36 | 5.81 | 6.58 | 4.85 | 6.18 | 5.52 |
| Kamalapur | 3.580 | 7.80 | 4.14 | 8.08 | 3.90 | 8.03 | 6.83 | 5.61 | 6.71 |
| Motijhil | 1.700 | 7.58 | 3.62 | 5.85 | 3.25 | 3.80 | 5.54 | 7.69 | 5.36 |
| Shampur | 0.959 | 7.70 | 6.07 | 8.30 | 2.02 | 7.25 | 7.63 | 3.68 | 6.81 |
| Uttarkhan | 1.980 | 6.67 | 3.84 | 7.54 | 2.33 | 6.49 | 5.68 | 7.64 | 5.09 |

^*^Municipal drinking water' refers to the water supplied by the city government

^§^Non-municipal water' includes water from private sources such as wells or boreholes
