# Supplementary material for: Variation in exposure in neighborhoods of Dhaka, Bangladesh across different environmental pathways: The influence of human behavior on fecal exposure in urban environments
Source: PLoS One. 2026 Jan 2;21(1):e0319883. doi: 10.1371/journal.pone.0319883 (PMC12758677; doi:10.1371/journal.pone.0319883)
Supplement: S7 Table — (DOCX) [file pone.0319883.s007.docx]

S7 Table: Comparing fecal exposure risks for children (age 5-12 years) by pathway and neighborhood characteristics

| **Pathway** | **^*^High-income with poor WASH.vs.high-income with improved WASH** | **Low-income vs. high-income with improved WASH** | **Low-income vs. high-income with poor WASH** |
| --- | --- | --- | --- |
| Open drain | <0.001 | <0.001 | <0.001 |
| Raw produce | 0.007 | 0.603 | 0.001 |
| Drinking water | 0.005 | <0.001 | 0.003 |
| Surface water | <0.001 | <0.001 | <0.001 |
| Floodwater | <0.001 | <0.001 | 0.01 |
| Latrine swab | 0.002 | <0.001 | <0.001 |
| Bathing water | 0.976 | 0.93 | 0.378 |
| Street food | <0.001 | <0.001 | 0.05 |
| Non- municipal water | <0.001 | <0.001 | 0.002 |

^*^P-values represent the results of the Chi-Square Test of independence comparing fecal exposure risks for children by pathway and neighborhood characteristics.
