# Supplementary material for: Variation in exposure in neighborhoods of Dhaka, Bangladesh across different environmental pathways: The influence of human behavior on fecal exposure in urban environments
Source: PLoS One. 2026 Jan 2;21(1):e0319883. doi: 10.1371/journal.pone.0319883 (PMC12758677; doi:10.1371/journal.pone.0319883)
Supplement: Attachment — Submitted filename: Response to reviewers.docx [file pone.0319883.s011.docx]

***Response to reviewers comments***

Aug 05, 2025

**Subject:** PONE-D-25-07134 authors’ response

Dear D. Daniel,

We sincerely thank you and the reviewers for your thoughtful and constructive feedback on our manuscript titled **“**Variation in exposure in neighborhoods of Dhaka, Bangladesh across different environmental pathways: the influence of human behavior on fecal exposure in urban environments (Manuscript ID: PONE-D-25-07134). We are grateful for the time and effort invested in reviewing our work.

We have carefully considered all comments and suggestions provided by the reviewers. We are pleased to confirm that we **agree with all the comments** and have addressed them accordingly in the revised version of the manuscript.

To summarize:

- We revised the manuscript to clarify key points as suggested.
- We improved the logical flow and addressed areas where additional explanation was needed.
- We incorporated the recommended edits to enhance clarity and scientific rigor.
- We have corrected the formatting and file name of the manuscript and supporting documents
- All grammatical and formatting issues raised have been corrected.

A point-by-point response to each comment is provided in the attached document for your review. Changes made to the manuscript are track changed/highlighted for ease of reference. A clean version of the manuscript is also attached.

We also added the “Funding Information” below which was missing:

*“The study was financially supported by the Bill & Melinda Gates Foundation (grant no. 00010161) through the Rollins School of Public Health at Emory University. icddr,b acknowledges with gratitude the commitment of the Bill & Melinda Gates Foundation and Rollins School of Public Health, Emory University to its research efforts. We acknowledge the World Bank Bangladesh Country Office team for their efforts in partnering, workshops, and contributing to sampling/study design. icddr,b is also grateful to the Governments of Bangladesh, and Canada for providing core/unrestricted support. The funders had no role in study design, data collection and analysis, decision to publish, or preparation of the manuscript.”*

We believe these revisions have significantly improved the quality and clarity of the manuscript. We hope that the revised version meets the expectations of the reviewers and the editorial board. Please do not hesitate to contact us if further clarification is required. We sincerely thank you again for the opportunity to revise and resubmit our work.

Sincerely,

**Dr. Nuhu Amin**

Associate Scientist, International Centre for Diarrhoeal Disease Research, Bangladesh

Email: nuhu.amin@icddrb.org

**Editor’s comments:**

**Dear Dr. Amin,

Thank you for submitting your manuscript to PLOS ONE. After careful consideration, we feel that it has merit but does not fully meet PLOS ONE’s publication criteria as it currently stands. Therefore, we invite you to submit a revised version of the manuscript that addresses the points raised during the review process.

Please submit your revised manuscript by Jul 04 2025 11:59PM. If you will need more time than this to complete your revisions, please reply to this message or contact the journal office at plosone@plos.org. When you're ready to submit your revision, log on to** [**https://www.editorialmanager.com/pone/**](https://www.editorialmanager.com/pone/) **and select the 'Submissions Needing Revision' folder to locate your manuscript file.**

**If applicable, we recommend that you deposit your laboratory protocols in protocols.io to enhance the reproducibility of your results. Protocols.io assigns your protocol its own identifier (DOI) so that it can be cited independently in the future. For instructions see:** [**https://journals.plos.org/plosone/s/submission-guidelines#loc-laboratory-protocols**](https://track.editorialmanager.com/CL0/https:%2F%2Fjournals.plos.org%2Fplosone%2Fs%2Fsubmission-guidelines%23loc-laboratory-protocols/1/010f0196ef84f38b-191c2112-b3be-47e9-8473-25ee7f6d935f-000000/T7k4dNLRQmhJ3W55hwbG0QqciqCtjrONSY7N4FW35Q4=212)**. Additionally, PLOS ONE offers an option for publishing peer-reviewed Lab Protocol articles, which describe protocols hosted on protocols.io. Read more information on sharing protocols at https://plos.org/protocols?utm_medium=editorial-email&utm_source=authorletters&utm_campaign=protocols.**

**Response:** We have included the protocols in the protocols.io with the DOI: 10.17504/protocols.io.q26g7nbe9lwz/v1.

We have put the information on page 5, lines 141-42: The laboratory protocol is available in the SaniPath website [1] and has also been uploaded to the protocols.io [2].

**Journal Requirements:**

**When submitting your revision, we need you to address these additional requirements.**

**1. Please ensure that your manuscript meets PLOS ONE's style requirements, including those for file naming. The PLOS ONE style templates can be found at**

**https://journals.plos.org/plosone/s/file?id=wjVg/PLOSOne_formatting_sample_main_body.pdf and**

**https://journals.plos.org/plosone/s/file?id=ba62/PLOSOne_formatting_sample_title_authors_affiliations.pdf**

**Response:** We have corrected the file naming and formatting

**2. We note that the grant information you provided in the ‘Funding Information’ and ‘Financial Disclosure’ sections do not match.**

**When you resubmit, please ensure that you provide the correct grant numbers for the awards you received for your study in the ‘Funding Information’ section.**

**Response:** We have checked the funding information, and all information is correct: The study was financially supported by the Bill & Melinda Gates Foundation (grant no. 00010161) to the Rollins School of Public Health at Emory University.

**3. When completing the data availability statement of the submission form, you indicated that you will make your data available on acceptance. We strongly recommend all authors decide on a data sharing plan before acceptance, as the process can be lengthy and hold up publication timelines. Please note that, though access restrictions are acceptable now, your entire data will need to be made freely accessible if your manuscript is accepted for publication. This policy applies to all data except where public deposition would breach compliance with the protocol approved by your research ethics board. If you are unable to adhere to our open data policy, please kindly revise your statement to explain your reasoning and we will seek the editor's input on an exemption. Please be assured that, once you have provided your new statement, the assessment of your exemption will not hold up the peer review process.**

**[Note: HTML markup is below. Please do not edit.]**

**Response:** The SaniPath data is available for download at <https://www.sanipath.net/data>

**Reviewers' comments:**

**Authors responses to Questions**

**Comments to the Author**

1. Is the manuscript technically sound, and do the data support the conclusions?

The manuscript must describe a technically sound piece of scientific research with data that supports the conclusions. Experiments must have been conducted rigorously, with appropriate controls, replication, and sample sizes. The conclusions must be drawn appropriately based on the data presented.

Reviewer #1: Yes

Reviewer #2: Partly

Reviewer #3: Partly

2. Has the statistical analysis been performed appropriately and rigorously?

Reviewer #1: I Don't Know

Reviewer #2: I Don't Know

Reviewer #3: I Don't Know

3. Have the authors made all data underlying the findings in their manuscript fully available?

The PLOS Data policy requires authors to make all data underlying the findings described in their manuscript fully available without restriction, with rare exception (please refer to the Data Availability Statement in the manuscript PDF file). The data should be provided as part of the manuscript or its supporting information, or deposited to a public repository. For example, in addition to summary statistics, the data points behind means, medians and variance measures should be available. If there are restrictions on publicly sharing data—e.g. participant privacy or use of data from a third party—those must be specified.

Reviewer #1: Yes

Reviewer #2: No

Reviewer #3: No

Response: The data has already been made freely available for download at https:// www.sanipath.net/data.

4. Is the manuscript presented in an intelligible fashion and written in standard English?

PLOS ONE does not copyedit accepted manuscripts, so the language in submitted articles must be clear, correct, and unambiguous. Any typographical or grammatical errors should be corrected at revision, so please note any specific errors here.

Reviewer #1: No

Reviewer #2: Yes

Reviewer #3: Yes

Review Comments to the Author

Please use the space provided to explain your answers to the questions above. You may also include additional comments for the author, including concerns about dual publication, research ethics, or publication ethics. (Please upload your review as an attachment if it exceeds 20,000 characters)

# Reviewer #1:

**The authors present an interesting study characterizing fecal pollution sources, pathways, and exposures across communities in Dhaka. Please expand on the methods and revise the conclusion. More information regarding the SaniPath Tool and its incorporation into the study is needed as well.**

**Response:** Thank you for your thoughtful comments and suggestions. We agree with your observations and have revised the manuscript to provide additional clarity on the SaniPath Tool, the methods, and the study's conclusions. Specifically, the following changes have been made:

**Introduction (Page 2, Lines 70–74):**
We have clarified the definition and functioning of the **SaniPath Tool**, emphasizing its role in assessing environmental fecal exposure through multiple pathways.

**Introduction (Page 3, Lines 90–96:**
We have explained how this study differs from the previous SaniPath study in Dhaka (Amin et al. 2019):
"Amin et al. (2019) conducted a cross-sectional study to assess the magnitude of fecal contamination in the environment across low-income, high-income, and transient/floating neighborhoods in urban Dhaka by measuring the frequency and concentration of E. coli [24]. However, the study did not assess or compare the levels of E. coli contamination with human exposure frequency by neighborhood. This highlights the need to integrate environmental contamination data with human exposure assessments to better understand health risks and prioritize interventions."

**Methods (Page 5, Lines 160–164:**
We have added information regarding laboratory quality assurance and quality control (QA/QC), including the use of field blanks, duplicates, and data sources and protocols.

“*For quality assurance, we included both a negative control and a field blank. The negative control consisted of PBS or sterile water processed in the lab using the same IDEXX methods, while the field blank involved PBS or sterile water taken to the field and "collected" using the same procedures as actual samples, to verify proper sample collection techniques”*

**Methods (Page 7, Lines 194–202):**
We have expanded the description of the questionnaire used for exposure data collection:
"*Questions about frequency of drinking water, ingestion of raw produce and street food, bathing, and using a communal/shared latrine were framed on a weekly timescale. Respondents were asked about consumption of municipal drinking water and non-municipal drinking water on a different frequency scale. The options given to respondents were “Every day in the past week”, “4 to 6 days in the past week”, “3 or less days in the past week”, “Never”, and “Do not know.” The reason for using different time scales is that drinking water is a common behavior that may be difficult to recall the number of times consumed in a week. Questions about frequency of contact with open drains, surface water, and flood water were framed on a monthly timescale as these contacts were likely to occur less often*."

**Methods (Page 8, Lines 252-258):**
We have clarified the steps of the exposure analysis:
"An exposure metric (𝐸) was calculated for each pathway as the log-transformed product of the estimated dose and the percentage of the population exposed. A pathway was classified as dominant if its 𝐸 value was greater than 10 (indicating high risk) or within one log unit of the maximum 𝐸 value observed (e.g., if 𝐸ₘₐₓ = 5, dominant pathways are those with 𝐸 ∈ 4 ≤E≤5). If all 𝐸 values were below 1, no dominant pathway was identified. In the absence of established thresholds for environmental pathways, these cutoffs were informed by formative and pilot testing conducted in Accra [18]."

We have also added website links within the citations and revised the conclusion section to better reflect these methodological clarifications and highlight the integration of exposure behavior and *E. coli* contamination data.

**Line 22: nine environmental pathways, not nine-environmental pathways**

**Response:** corrected throughout the manuscript (see changes in Line 29)

Line 23: Does the SaniPath tool need at trademark?

**Response:** Thank you for your suggestion. We put trademark “SaniPath™” on first mention the manuscript. See changed on page 1, line 30 and page 2, line 70

**Line 66: Please expand a bit on the SaniPath tool**

**Response:** We have added more information on lines 70-74 to describe the SaniPath tool.  We also add a citation of Raj et al 2020 tool paper and a link to the SaniPath website.

“*The SaniPath^TM^ Exposure Assessment Tool has been implemented in several cities in South Asia and Sub-Saharan Africa (Wang, et al 2017), and includes customized primary data collection on fecal contamination of the residential environment and human exposure behavior and automated Bayesian analyses to estimate risk of fecal exposure [18, 19]*.”

**Line 93: Can you list the date of this meeting?**

**Response:** Yes, we have updated the manuscript to include the date (February 14, 2017). See the date on page 3, line 107.

**Data collection: Did you have any controls for the microbial testing?**

**Response:** We did not include a positive control, but we included both a negative control and a field blank. Negative control being a PBS/sterile water that was processed using the same IDEXX methods in the lab and a field blank, meaning PBS/sterile water that was taken to the field and "collected" using the same techniques, to ensure proper sample collection techniques.
See changes on page 5, lines160-164.

**Line 164: Please remove "like the mean".**

**Response:** Removed (Line 238)

**Define JAGS**

**Response:** JAGS refers to “Just Another Gibbs Sampler”. We have added this in the manuscript on page 8, line 240.

**Line 169: Please include references for the "existing literature and formative study data". Is there a table describing this data?**

**Response:** Thank you for your careful review. We have added relevant references. A table describing this data is included in the appendix of the methods paper for the SaniPath Tool by Raj et al, S2. Appendix https://doi.org/10.1371/journal.pone.0234364.s002. (Changes in line 244 and lines 270-277)

[18]. Raj SJ, Wang Y, Yakubu H, Robb K, Siesel C, Green J, et al. The SaniPath Exposure Assessment Tool: A quantitative approach for assessing exposure to fecal contamination through multiple pathways in low resource urban settlements. PLoS One. 2020;15(6):e0234364.

[24]. Wang Y, Mairinger W, Raj SJ, Yakubu H, Siesel C, Green J, et al. Quantitative assessment of exposure to fecal contamination in urban environment across nine cities in low-income and lower-middle-income countries and a city in the United States. Science of The Total Environment. 2022;806:151273. doi: <https://doi.org/10.1016/j.scitotenv.2021.151273>.

[30]. Amin N, Rahman M, Raj S, Ali S, Green J, Das S, et al. Quantitative assessment of fecal contamination in multiple environmental sample types in urban communities in Dhaka, Bangladesh using SaniPath microbial approach. PLoS One. 2019;14(12):e0221193.

**You also repeat the exposure assessment methodology in lines 169-171. Please remove.**

**Response:** Thanks for your careful review. We have revised the section. See changes on page 8, lines 243-250

**The second half of the paragraph is repeated. Please revise.**

**Response:** We have revised the whole section to avoid repetition. See changes in lines 243-260.

**Data analysis: additional information is needed here. Are there any equations for dose? More information on the software is needed too.**

**Response:** We have added details on the analysis plan including the tools used for the analysis. The dose we used for the SaniPath tool is in the SaniPath Tool paper S2. Appendix https://doi.org/10.1371/journal.pone.0234364.s002. (See changes in lines 252-260.

**There is some information in the footnotes under Table 2, but this needs to be included in the methods section.**

**Response:** We have added more information about the behavior questions to the methods section on lines 293-310. We also added more detailed information in the results sections.

**The Results section has several typos and the figures are very blurry and hard to review. I had trouble accurately assessing the results given the quality of the figures.**

**Response:** Thanks for checking, we have checked all typos throughout the manuscripts and improved the quality/resolution of the figures.

**Line 412: Please include references for "and studies by others".**

**Response:** References added in line 504.
*Fecal contamination of produce and street food in Bangladesh and other LMICs has been reported in numerous SaniPath deployments [21] and studies by others [13, 36, 37].*

**Line 591: Is there a typo for SFD.**

**Response:** we have checked the spelling and there is no typo. “SFD” is the conventional acronym for “Shit Flow Diagram” Please check here: <https://sfd.susana.org/> (Line 701)

**The conclusion is very long and much of the information discussed should be included in the discussion. Please revise.**

**Response:** Thank you for this thoughtful suggestion. We understand your concern regarding the length of the conclusion. However, as the study covers multiple exposure pathways and neighborhood contexts, we aimed to provide distinct and practical recommendations to inform future interventions and policy actions.

We have carefully reviewed and revised the conclusion to make it more concise and focused, while still retaining the key messages that emerged from our findings. Given the complexity and breadth of the study, a certain level of detail remains necessary to ensure clarity and utility for public health practitioners and decision-makers. We hope the revised version strikes a better balance between brevity and completeness. Thank you again for your feedback.

# Reviewer #2:

**Overall comments:
The authors investigated fecal exposure from different routes in urban Dhaka using a SaniPath methodology. There are some places where the manuscript could be strengthened, outlined in detail below.**

**Response:** We sincerely thank the reviewer for the thoughtful feedback. We appreciate your recognition of our work. We have carefully addressed all the detailed comments provided and revised the manuscript accordingly to improve its clarity, structure, and overall quality. We hope the revised version meets your expectations.

**Specific comments:
Abstract
It’s unclear to me what the %’s reported in the results section of the abstract mean. Please provide a more detailed description of this.**

**Response:** We have clarified the sentence (Line 43): Children experienced higher estimated monthly fecal exposure doses than adults, primarily through ingestion of contaminated municipal water (all community average=59.2% of population is exposed, either adults or children).

**Introduction**
**I’m not entirely clear what the contribution of this paper is compared to a similar paper that was previously published by the same authors from this data (Amin et al. 2019). There seems to be a fair amount of overlap in the methods and discussion sections, with both papers presenting relatively similar take-aways. It would be helpful if the specific results from that paper were outlined in the introduction to explain the specific knowledge gap left by that paper that this paper aims to fill.**

**Response**: Thank you for this important comment. Amin et al. 2019 presents the data on fecal contamination in the environmental samples from this assessment. However, exposure (and ultimately health risks from exposure) depends on two parameters - fecal contamination in these different environmental compartments and contact (or ingestion) with the environment. This paper presents the full results of the exposure assessment that considers both the magnitude of fecal contamination in the environment and the frequency of human behavior that results in direct or indirect ingestion of fecal indicators in the environment. We have modified **lines 90-96** to clarify this distinction and the unique contribution of this paper.

**Methods
The abstract says that 900 environmental samples were collected and analyzed for E.coli whereas the methods sections instead says that 1000 environmental samples were collected and analyzed. Please clarify this discrepancy.**

**Response:** We have corrected the inconsistency. The actual environmental samples were 1000. (Line 32)

**The methods specify that data was collected to understand and compare exposure between adults (>18 years old) and children (5–12 years old). Please clarify and provide justification in the manuscript as to why children <5 years old were not included in this study. Children <5 are typically considered an especially vulnerable population for health concerns related to fecal contamination exposure.**

**Response:** We have added some clarification to the text.
*“The Tool focuses on exposure to fecal contamination in public, not private, spaces for adults and children ages 5–12. This focus was chosen because public areas are more influenced by sanitation policies, often drive private contamination, are where most environmental contact occurs in crowded urban settings, and allow for quicker data collection (Lines174-184). It does not assess exposure for children under 5, who typically spend more time in private spaces with caregivers (Lines 172-182).”*

**Lines 169-176 seem to be a repeat of what you said in lines 163-169 in slightly different words. Please review and rectify.**

**Response:** We have removed the repetition in this section (See changes: Lines 243-250)

**Were questions asked about whether the respondent did anything to treat the water before drinking it? Or whether they cleaned the raw produce before eating it? These types of practices could change exposure, so it would be helpful to clarify these points.**

**Response:** Yes, we asked about water treatment and produce washing, but these were not included in exposure modeling. Evidence shows produce washing alone has limited impact on *E. coli* removal—especially without added sanitizers—underscoring its potential importance in exposure assessments. We clearly stated this in our limitations and recommend incorporating these behaviors in future analyses. See changes on page 7, lines 204-208 and lines 611-619.

**What assumptions were used for calculating the amount ingested from contact with things like drinking water, surface water, flood water, etc.? These assumptions play a key role in estimating and comparing exposure among the different pathways that underlie your results and conclusions, and it’s not clear to me from the methods section what data was used to estimate ingestion/exposure based on reported frequency of contact with each.**

**Response**: We briefly explained the key assumptions made and added the reference to the details of assumptions published for the SaniPath Tool assessment (Lines 270-277).

*“The key assumptions for ingestion amounts from different environmental pathways are that intake volumes and durations of exposure are fixed values rather than distributions due to limited literature on their variation. The SaniPath tool uses age-specific parameters (adults vs. children) and pathway-specific multiplication factors to calculate the volume or percentage consumed per exposure event. For water-related pathways, ingestion is calculated using the formula: (Intake Volume/Intake Time) × Duration of Event, while food and surface contact pathways use fixed percentages of consumption per event. The details of those assumptions can be found at the S2 Appendix of Raj et al. (2020) [18].”*

**Please update the methods section to include more information about the survey conducted, such as who performed the survey, language of survey, and relevant training of enumerators. Details of who collected the environment sample should also be included.**

**Response:** Thank you for your comment. We have updated the methods section to include the following details (Lines 134-145):

*“Two separate teams of trained field staff conducted the data collection activities. Key informant interviews, mapping and environmental samples were collected by a team of fieldworkers from icddr,b, led by the lead author (NA). Behavioral surveys were conducted by a local NGO, DATA, under the supervision of the Emory University team (SR). All data were collected using mobile phones programmed with ODK (Open Data Kit). The environmental sampling forms were administered in English, while the behavioral survey questionnaires were developed in both English and Bengali to ensure cultural and linguistic appropriateness. Both teams received standardized training conducted jointly by the icddr,b and Emory teams in Dhaka. The training covered study objectives, ethical considerations, proper use of ODK tools, sample collection protocols, and quality control procedures to ensure consistency and reliability in data collection across all sites.”*

**Results
At the beginning of the results section (e.g., lines 197-212 such as “Household behavioral surveys suggested that children in floating and low-income neighborhoods reported more frequent contact (i.e., at least one contact or ingestion event) with nearly all environmental pathways”), it is not clear to me what the time period for these contacts is – one contact per day, week, etc? Please specify this more clearly throughout.**

**Response:** Thank you for pointing this out. We agree that the time frame for reported exposures should be stated more clearly to avoid confusion.

We have revised the relevant sections in the Results and Methods to clarify that exposure and ingestion frequencies were reported on a weekly basis for all pathways, except for contact with drain water, surface water, and floodwater, which were reported on a monthly basis due to their less frequent occurrence. These time frames have now been consistently specified throughout the manuscript to improve clarity and accuracy in interpreting the exposure data (See changes: Lines 293-310).

**From Figure 1, the exposure pathways seem relatively neighborhood specific. I suggest providing more discussion of this among the manuscript.**

**Response:** We have added following sentences about fig. 1 in the discussion section, lines 458-462.

*“The dominant exposure pathways varied across neighborhoods in Dhaka. However, slums within the same geographic region (north vs. south) revealed similar patterns in dominant pathways, likely driven by comparable sanitation infrastructure. In contrast, non-slum neighborhoods within the same regions showed distinct dominant pathways with no clear geographic clustering pattern”.*

**The text on Figure 3 is difficult to read and I would recommend using a higher resolution for this image.**

**Response:** We have improved the quality of the graphs.

**From Figure 3, it seems that many pathways are important sources of high levels of fecal exposure. Given this figure, I’m not entirely convinced about some of the dominant pathway findings between children/adults or northern/southern areas since many pathways seem to lead to high levels of exposure and need to be addressed. For example, lines 294-301 and 303-313 are not entirely convincing based Fig 3. I suggest providing stronger justification for these points, or revising the take-aways. For example, were any statistical tests done to support the conclusions in results about different among different neighborhoods, adults/child, etc?**

**Response:** We appreciate your careful review. Living in a contaminated environment such as slums in Dhaka exposes residents to fecal contamination through many different pathways. Usually, multiple pathways can lead to high level of fecal exposure. While SaniPath exposure assessment provides the full picture of exposure from all the pathways by neighborhood, it also summarizes the results to prioritize and guide public health investment for the pathways that make the “dominant” (more than 2 log of magnitude in average exposure) contribution to the total fecal exposure. As the reviewer pointed out, in some neighborhoods, multiple pathways had high levels of exposure. For example, from Figure 3, you could see many pathways for Gabtoli had exposure levels larger than 10^6 MPN *E. coli*/month. Therefore, you see surface water, street food, produce, and flood all make great contributions to the total exposure in Figure 2. In such a case, we identified all of them as dominant pathways that need to be considered for public health interventions and investments. On the other hand, in Gulshan, the raw produce pathway is responsible for exposure larger than 10^7 MPN *E. coli*/month and contributes to 99% of the total exposure, which makes this the dominant pathway in this neighborhood.

We did conducted hypothesis testing for behavior survey data (Table S7 and S8) and environmental sample contamination level data (Amin et al. 2019). However, the exposure estimates are from Monte Carlo simulations instead of being directly measured. Therefore, we could not perform hypothesis testing for the simulation outcomes from the Monte Carlo simulation. In Raj et al. (2020), we conducted a validation study and reported: “While the accuracy of the Tool may not allow us to detect small differences between different pathways or neighborhoods (less than 1 log_10_ average dose), the methods can be used to quantify and compare the relative contribution of these environmental pathways to the total exposure to fecal contamination.”

**Lines 350-351: “10310^3103 MPN” is unclear, please rectify.**

**Response:** Sorry, this was a typo. We corrected the digits to 10^3^. (line 437)

**It’s not clear exactly what poor WASH vs improved WASH means in this context when you present results comparing the two. Can you provide more details about what you mean by both?**

**Response**: Thank you for this helpful observation. We agree that the distinction between poor (unimproved) and improved WASH conditions should be clearly defined for readers. In response, we have revised the Methods section to include an operational definition used in this study.

While we drew upon the WHO/UNICEF Joint Monitoring Programme (JMP) classification as a reference, our definitions were adapted based on the observed infrastructure and access conditions in the study neighborhoods.

Specifically, unimproved WASH refers to the absence or inadequacy of safe water on premises (as observed in floating and low-income communities), lack of sanitation with proper fecal sludge management, and limited hygiene. These areas are typically characterised by open drains, and shared or non-functional latrines.

In contrast, improved WASH refers to conditions in communities with piped water supply on premises and sanitation systems connected to a sewer or covered drainage network (as typically observed in high-income communities).

This operational distinction has been added to the revised manuscript (Methods and result section- lines 114-121; lines 265-266; lines 380-388) and is consistently used throughout the analysis.

**Was any information collected about household characteristics that could be added to help interpret and contextualize the findings?**

**Response:** Thank you for this comment. We did not collect any additional household characteristic data as a part of this assessment. Since the primary focus of this assessment was on the public domain, we were less focused on the contribution of household-level fecal contamination to an individual’s exposure. We acknowledge that household-level exposure can contribute to poor public health as well, but our focus on the public domain was driven by where city or government interventions would most likely have impact. We have done past studies that incorporated household level exposures in Ghana (See Wang et al 2017 https://pmc.ncbi.nlm.nih.gov/articles/PMC5637579/) and India (Berendes et al., 2017 Am. J. Trop. Med. Hyg., 96(6), 2017, pp. 1404–1414

Adding additional household characteristics could be a consideration for future assessments.

**Discussion
Can the estimated values of Ecoli ingested be put into context of diarrhea risk? This would help interpret the findings of logs of Ecoli ingested for different pathways presented in the results section.**

**Response:** Thank you for this insightful comment. We agree that linking fecal exposure to health outcomes such as diarrhea risk would enhance interpretation. However, the estimated exposure to *E. coli*, a fecal indicator bacteria, cannot be directly translated into diarrhea risk due to several limitations in establishing a quantitative dose–response relationship for *E. coli*.

As highlighted in Section 4.3 of Wang et al. (2022) [DOI: 10.1016/j.scitotenv.2021.151273], exposure to fecal contamination has been proposed as a useful endpoint in its own right (Wang et al., 2017; Ngure et al., 2013; Mattioli et al., 2015). The SaniPath Exposure Assessment Tool—which forms the basis of our analysis—is designed to evaluate human exposure to fecal contamination across multiple environmental pathways. This exposure is a necessary prerequisite for enteric infection and illness, but not a direct measure of disease burden.

While a benchmark threshold for *E. coli* ingestion that corresponds to diarrhea risk does not currently exist, higher levels of fecal exposure are considered indicative of increased probability of ingesting enteric pathogens and consequently a greater risk of infection and illness. Therefore, the estimated *E. coli* ingestion values are best interpreted as relative indicators of exposure risk across pathways and communities, rather than predictors of specific health outcomes.

**Supporting data
The authors mentioned that “All relevant data are within the manuscript and its Supporting Information files.”, but I did not see the underlying data submitted with the SI files.**

**Response:** The data has already been made freely available for download at https:// www.sanipath.net/data.

# Reviewer #3:

**The authors are reporting environmental and behavioral data collected and analyzed via the SaniPath tool to assess human exposure to fecal indicator bacteria across several pathways in Dhaka, Bangladesh. Their work provides data that can inform local risk management strategies to reduce exposure to fecal contamination in Dhaka. Please see below for further comments and suggestions:

My biggest source of confusion is the aims. “Risks of exposure” and “exposure risks” are unclear and uninformative phrases that are inconsistent with traditional microbial risk assessment approaches. Are the authors estimating exposure (e.g., via a dose) or are they estimating a risk (e.g., of a non-cancer health endpoint, like an infection). Similarly, “exposure behavior” is not clear to this reader what is being assessed.**

**Response:** The SaniPath tool estimates the average dose at a population level within a neighborhood, which is the average number of *E. coli* ingested per person per month. We agree that the use of “risk of exposure” is not appropriate and replaced it as “exposure” with definition added to the method section (Line 30, 385, 412, 451, 520, 590). We also revised the “exposure behavior” as “contact behavior that leads to exposure” (Line 73, 99, 179). We also provide definition in lines 258-260.

**Most of the analysis methods for this paper were completed by the SaniPath Exposure Assessment tool, whose methods were described in another paper. As a result, this paper is not easy to understand without reading the other paper. I suggest the authors BRIEFLY describe the key inputs and data processing steps of the tool.**

**Response:** This comment is consistent with the first comment from Reviewer 1, which we have addressed in detail. We kindly request the reviewer to refer to our response to that section for further clarification.

**Although they have provided numerous data tables in the supplement, the authors have not made a cleaned or de-identified dataset available.**

**Response:** The data set is available on the SaniPath website https://www.sanipath.net/data.

**Line 129-138: Please explain the difference between household vs. community vs. school surveys. Was there any difference in content between the different versions of the surveys. Were participants compensated for time they gave to complete surveys?**

**Response:** There was no difference in content of the survey questionnaire across the three respondent groups. However, the focus of each survey type varied slightly based on the setting. Household surveys were conducted to understand individual-level exposure behaviors. Community surveys focused on various components of community-level exposures. School surveys aimed to explore exposure pathways specific to school-age children. No participants were compensated for their time in completing the surveys. More information is added on lines 176-182.

**166 - What is JAGS? Please explain/define.**

**Response:** **JAGS** (Just Another Gibbs Sampler) is a program for Bayesian statistical analysis using Markov Chain Monte Carlo (MCMC) simulation. It allows users to define complex hierarchical models using a BUGS-like syntax and is particularly useful for estimating posterior distributions in Bayesian inference. JAGS is platform-independent and often used in combination with R via the RJAGS package for model fitting and the coda package for analyzing MCMC outputs. It supports both discrete and continuous variables, missing data, and is widely applied in fields such as epidemiology, ecology, and environmental health. We have added the abbreviation and citation [29] to clarify the software in line 240.

**Lines 169-178. More in text description of exposure and risk methodology is encouraged. As is, it is not clear what equations were used to estimate the dose and risk. In addition, if the authors are providing a risk, they should clarify what the outcome measure is (i.e., hazard quotient). Please explain.**

**Response:** We have added detailed analysis information including the tools used for the analysis. We are estimating exposure not risk (See changes in the data analysis section).

**Line 315. Could you provide more details about how the doses were estimated? I presume dose was a combination of a level of e.coli and a model that incorporates differences in ingestion rates and other behaviors. Given that the reader does not have access to the underlying inputs of e.coli and behavior, the reader cannot ascertain the extent to which the differences in dose were attributable to the level of e.coli or the behavior. Please provide a sensitivity analysis for thiss.**

**Response:** Thank you for your comments. We do not think a sensitivity analysis is necessary for this study. We have conducted a series of validation studies for the SaniPath tool as described in Raj et al. 2020.The dose assumptions we used for the SaniPath tool are in the SaniPath Tool paper S2. Appendix <https://doi.org/10.1371/journal.pone.0234364.s002>. (also see revised analysis description on page 9).

**Line 296. Please describe “improved wash infrastructure”**

**Response:** We have addressed this comment by providing the operational definitions, as discussed in response to Reviewer 2's comments. This operational distinction has been added to the revised manuscript (Methods and result section- lines 114-121; lines 265-266; lines 380-388) and is consistently used throughout the analysis.

**Line 334 - How was the significant difference determined? If it was determined statistically, these methods need to be described in the manuscript and results of hypothesis testing provided. This comment applies throughout the manuscript when assertions of differences are described.**

**Response:** We agree with you and removed the word “significant” while reporting results.

**Line 364. The phrase “risk of exposure” is not clear. Please rephrase and present risk estimates that relate to a health outcome (e.g., risk of infection or symptoms). This would provide more actionable information to inform risk mitigation strategies.**

**Response:** This has been answered with your earlier comment.

The SaniPath tool estimates the average dose at a population level within a neighborhood, which is the average number of *E. coli* ingested per person per month. We agree that the use of “risk of exposure” is not appropriate and replaced it as “exposure” with definition added to the method section (Line 30, 385, 412, 451, 520, 590). We also revised the “exposure behavior” as “contact behavior that leads to exposure” (Line 73, 99, 179). We also provide definition in lines 258-260.


**Line 570 - What does icddr,b mean? This is only written out in author affiliations. Needs to be explained at first mention in MS.**

**Response:** We have elaborated icddr,b: International Center for Diarrheal Disease Research, Bangladesh (line 137).
