# Supplementary material for: Variation in exposure in neighborhoods of Dhaka, Bangladesh across different environmental pathways: The influence of human behavior on fecal exposure in urban environments
Source: PLoS One. 2026 Jan 2;21(1):e0319883. doi: 10.1371/journal.pone.0319883 (PMC12758677; doi:10.1371/journal.pone.0319883)
Supplement: Attachment — Submitted filename: Response to reviewers_30thOct.docx [file pone.0319883.s012.docx]

***Response to reviewers comments***

# Reviewer #2:

**The authors have adequately addressed most of my previous comments. I just have two comments remaining:**

*1) I remain confused about the intention of the different behavioral surveys mentioned on page 5. For example, I do not understand why household surveys were conducted with mothers of children <5 years when children <5 were not being assessed for exposure. If these surveys were used for estimating exposure of adults and children aged 5-12 years, why were households with children 5-12 years not targeted instead? And why did school surveys primarily focus on children aged 10-12 instead of children aged 5-12? More largely, it is not clear what information was obtained from these two surveys and how that data was used in combination with the community surveys to estimate exposure.*

**Response:** Thank you for the questions. Household surveys were conducted and analyzed for children ages 5–12, not for children under 5, and we have corrected this error. School surveys were administered to school-age children (ages 10–12), as this falls within the 5–12 age range and is appropriate for a classroom setting. Older children are also better able to understand the questions and select accurate responses. All surveys were standardized, and behavioral data were combined for the exposure assessment (i.e., adult data from all three survey types were aggregated, and child data from all three survey types were aggregated). Because the SaniPath assessment is designed to be a relatively rapid data collection exercise, providing three survey options—household, school, and community—allows users to either collect more data overall or select the survey types that best match their capacity. Collecting multiple survey types also helps overcome the limitations of accessing specific populations (e.g., high-income households may be harder to reach through household surveys). Using data from all three surveys ultimately increases the amount of information available to estimate exposure. ***Please find the changes on Page 6, Lines 168-178)***

***2)*** *Please carefully check the footnote edits and associated labels that have been changed for Table 2. There is a c in front of the Household surveys label that appears to be an error, and it also looks like the footnote for municipal and non-municipal water is no longer labeled after the relevant text in the table.*

**Response:** Thank you for your careful review. We have checked all footnotes and corrected accordingly.

# Reviewer #3:

*Most of the analysis methods for this paper were completed by the SaniPath Exposure Assessment tool, whose methods were described in another paper. As a result, this paper is not easy to understand without reading the other paper. I suggest the authors BRIEFLY describe the key inputs and data processing steps of the tool.*

*Response: This comment is consistent with the first comment from Reviewer 1, which we have addressed in detail. We kindly request the reviewer to refer to our response to that section for further clarification.*

*It is difficult to determine of Reviewer 1’s comments and your response is responsive to this suggestion. Please clarify. Note: The information provided on this website:* [*https://www.sanipath.net/sanipath-approach*](https://www.sanipath.net/sanipath-approach)

*Is the type of brief summary I was looking for. A figure/process diagram would also be helpful to show the inputs, processes and outputs of “the tool.” All three are necessary to understand what the tool is and how the collected data is used.*

**Response:** Thank you for your further comment. We have provided a lot of writing to BRIEFLY describe the analysis method in this paper (see line 261 and figure 1). We have added a diagram (Fig 1) and revised the method to incorporate this diagram.

*Line 315. Could you provide more details about how the doses were estimated? I presume dose was a combination of a level of e.coli and a model that incorporates differences in ingestion rates and other behaviors. Given that the reader does not have access to the underlying inputs of e.coli and behavior, the reader cannot ascertain the extent to which the differences in dose were attributable to the level of e.coli or the behavior. Please provide a sensitivity analysis for thiss.*

*Response: Thank you for your comments. We do not think a sensitivity analysis is necessary for this study. We have conducted a series of validation studies for the SaniPath tool as described in Raj et al. 2020.The dose assumptions we used for the SaniPath tool are in the SaniPath Tool paper S2. Appendix https://doi.org/10.1371/journal.pone.0234364.s002. (also see revised analysis description on page 9)*

*Validation and sensitivity analyses are different things – and the authors have not provided a defensible rationale for why the sensitivity analysis is not necessary (i.e., conducting a validation study is not appropriate). A validation study seeks confirms that the tool is accurately and reliably measuring what it is purported to measure. A sensitivity analysis assesses the relative uncertainty and assesses how changes in each input variable impact the output. Please address.*

**Response:** As to sensitivity analysis, we assume this is not the objective for this paper to do that. The objective of this paper is to use an established analtytical method to conduct exposure assessment in Dhaka not to justify or develop the SaniPath exposure assessment method.

The SaniPath tool uses relatively naive assumptions for intake volume (constant volume or percent based on best literature we can find). Please see the table below:

**Table 1.** Assumptions and factors used in the SaniPath Tool exposure model

1.          U.S. EPA. Exposure Factors Handbook 2011 Edition (Final Report). 2011.

2.          SaniPath. SaniPath Phase 1 Data.

3.          U.S. EPA. *Descriptive Statistics from a Detailed Analysis of the National Human Activity Pattern Survey (NHAPS) Responses*. Washington, DC; 1996. doi:EPA/600/R-96/148

4.          Dorevitch S, Panthi S, Huang Y, et al. Water ingestion during water recreation. *Water Res*. 2011;45(5):2020-2028. doi:10.1016/j.watres.2010.12.006

5.          Dufour AP, Evans O, Behymer TD, Cantú R. Water ingestion during swimming activities in a pool: a pilot study. *J Water Health*. 2006;4(4):425-430. <http://www.ncbi.nlm.nih.gov/pubmed/17176813>. Accessed October 16, 2019.

6.          Percent consumed estimate is based on serving size of produce or street food that an adult could be expected to eat. For child estimates, the serving size is estimated to be half that of the adult portion.

In this table, you can see the "multiplication factor" between adults and children are within a difference of 1 log10 scale, except flood water and drain water (within a log10 scale difference of 1.22). Therefore, the difference in exposure between adults and children could be attributed to the intake assumption up to around 1 log10 difference (not a huge impact). For comparing different pathways, the largest difference of multiplication factor is around 4.2 log10 difference (drinking water vs. drain water). Although the number seems to be large, the idea that a person may drink 10,000 times more volume of drinking water compared to drain water seems to be reasonable. We agree that a sensitivity analysis can be helpful in a Monte Carlo simulation to examine the impact of different parameters and assumptions on the results. Since all the intake volumes are constant multiplication factors, making a linear relationship with exposure (e.g., making intake volume 10 times larger will lead to 10 times larger exposure). Given the very limited information (especially in variation) about intake volume available, we are not sure how useful the results of sensitivity analysis.
